# Involvement of Inositol Biosynthesis and Nitric Oxide in the Mediation of UV-B Induced Oxidative Stress

**DOI:** 10.3389/fpls.2016.00430

**Published:** 2016-04-12

**Authors:** Dmytro I. Lytvyn, Cécile Raynaud, Alla I. Yemets, Catherine Bergounioux, Yaroslav B. Blume

**Affiliations:** ^1^Department of Genomics and Molecular Biotechnology, Institute of Food Biotechnology and Genomics, National Academy of Sciences of UkraineKyiv, Ukraine; ^2^Laboratory of Cell Cycle Chromatin and Development, Institute of Plant Sciences Paris-Saclay IPS2, CNRS 9213, INRA 1403, Université Paris-Sud, Université Evry Val d’Essonne, Université Paris Diderot, Sorbonne Paris-Cite, Universite Paris-SaclayOrsay, France

**Keywords:** oxidative stress, nitric oxide, inositol-3-phosphate synthase 1, sodium nitroprusside, reduction–oxidation-sensitive green fluorescent protein 2, glutathione; oxidized disulfide form of glutathione

## Abstract

The involvement of NO-signaling in ultraviolet B (UV-B) induced oxidative stress (OS) in plants is an open question. Inositol biosynthesis contributes to numerous cellular functions, including the regulation of plants tolerance to stress. This work reveals the involvement of inositol-3-phosphate synthase 1 (IPS1), a key enzyme for biosynthesis of *myo*-inositol and its derivatives, in the response to NO-dependent OS in *Arabidopsis.* Homozygous mutants deficient for IPS1 (*atips1*) and wild-type plants were transformed with a reduction- *grx1-rogfp2* and used for the dynamic measurement of UV-B-induced and SNP (sodium nitroprusside)-mediated oxidative stresses by confocal microscopy. *atips1* mutants displayed greater tissue-specific resistance to the action of UV-B than the wild type. SNP can act both as an oxidant or repairer depending on the applied concentration, but mutant plants were more tolerant than the wild type to nitrosative effects of high concentration of SNP. Additionally, pretreatment with low concentrations of SNP (10, 100 μM) before UV-B irradiation resulted in a tissue-specific protective effect that was enhanced in *atips1*. We conclude that the interplay between nitric oxide and inositol signaling can be involved in the mediation of UV-B-initiated oxidative stress in the plant cell.

## Introduction

Ultraviolet B (280–315 nm waveband of the solar irradiation) amounts to almost 2% of short-wave radiation reaching living organisms in most ecosystems, and this proportion is likely to increase due to the ozone layer depletion (Caldwell et al., 2003; McKenzie et al., 2011). UV-B overexposure leads to numerous harmful consequences in plant cells by damaging DNA (cyclobutane pyrimidine and pyrimidine (6-4) pyrimidinone dimers formation), the photosynthetic apparatus (thylakoid disruption and disintegration of the double membrane envelope surrounding the chloroplast, destruction of chlorophyll and carotenoids) and membranes that undergo lipid peroxidation. Ultimately, these cellular damages can lead to programmed cell death (Lytvyn et al., 2010; Krasylenko et al., 2013), reduced growth and productivity and genomic instability (Kunz et al., 2006; Kataria et al., 2014). Interestingly, UV-B exposure can also improve plant resistance to pathogens (Paul, 2000; Kunz et al., 2006), probably due to the activation of defense mechanisms, which provides evidence for the activation of signaling cascades by UV-B exposure. Protective mechanisms induced by UV-B include the activation of secondary metabolism and more specifically the biosynthesis of phenylpropanoids and UV-B-protective carotenoids and flavonoids (Hollósy, 2002; Kovács and Keresztes, 2002; Frohnmeyer and Staiger, 2003).

Overproduction of ROS, namely, superoxide radicals, singlet oxygen, hydrogen peroxide, and hydroxyl radicals, caused by UV-B exposure, have both damaging actions and signaling functions in plant cell (Gill and Tuteja, 2010; Hideg et al., 2013; Kataria et al., 2014). Indeed, accumulation of ROS leads to OS activates defense reactions such as synthesis of UV-absorbing phenolic compounds; activation of DNA repair mechanisms; activation of enzymatic and non-enzymatic ROS scavenging; and overexpression of UV-B sensitive oxidative defense genes (Hideg et al., 2013; Kataria et al., 2014). Despite the considerable role attributed to ROS in plant cells response to UV-B, other signaling mediators leading to the activation of cellular protective mechanisms are predicted to exist. Indeed, treatment of *Arabidopsis thaliana* plants with the NO scavenger 2-phenyl-4,4,5,5-tetramethylimidazolin-L-oxyl-3-oxide (PTIO) and/or with an inhibitor of NO synthase [N^G^ -monomethyl-L-arginine (L-NAME)] have been shown to prevent UV-B induced expression of the flavonoid biosynthesis enzyme chalcone synthase (*CHS*). This observation indicates that up-regulation of *CHS* by UV-B requires NO (Mackerness et al., 2001), even though the existence of NO synthase in plants is still an open question (Corpas et al., 2004). In the past decades, NO has been recognized as a messenger molecule in plants that is involved both in physiological processes and stress responses (for detailed reviews see; Wendehenne et al., 2004; Yamasaki, 2005; Arasimowicz and Floryszak-Wieczorek, 2007; Besson-Bard et al., 2008; Siddiqui et al., 2011). Notably, a number of reports provide evidence for the protective effects of NO in plants challenged with UV-B. These effects include UV-B mediated changes of NO synthase-like and nitrate reductase activity; protective action of exogenous NO donors; interrelation of NO and ROS signaling pathways; NO impact on the ROS scavenging system activity; and the role of NO in UV-B perception by plant cell (summarized in Yemets et al., 2015).

Moreover, NO signaling has been implicated in many fundamental cellular processes (photosynthesis, organelles motility, hypersensitive response and programmed cell death) and cross-talks have been identified between NO-signaling and numerous pathways such as cytosolic calcium signaling; cyclic adenosine diphosphate ribose and cyclic guanosine 5′-monophosphate; salicylic, jasmonic acids and ethylene; ROS signaling, namely hydrogen peroxide; and MAPK-, salicylic acid-induced protein kinases (Yemets et al., 2015). Promising but totally undiscovered is the interrelation between NO and metabolic/signaling pathways related to *myo*-inositol derivatives. *Myo*-inositol derivatives play a critical role in eukaryotic cells as membrane structural lipids precursors and as signaling molecules. In plants these compounds are involved in a large number of cellular processes such as biogenesis of the cell wall and membrane structures, phosphate storage, cell signaling and cell resistance to external stressful factors (Loewus and Murthy, 2000). For example, *myo*-inositol participates to plant tolerance to salt and cold stress (Valluru and Van den Ende, 2011). Furthermore, *Arabidopsis atips1* mutants which are deficient for the 1L-*myo*-inositol-1-phosphate synthase (*AtIPS1*, E.C.5.5.1.4) and accumulate less than 10% of wild-type *myo*-inositol levels show light-dependent spontaneous cell death, and constitutive activation of biotic stress response genes (Meng et al., 2009; Bruggeman et al., 2014), providing further evidence for the role of *myo*-inositol or its derivatives in plant stress response. Some studies performed on animal models suggest that direct cross-talks may exist between UV-B and *myo*-inositol signaling. Indeed, inositol hexaphosphate was shown to inhibit UV-B induced activation of the transcription factors AP-1 and NF-κB, thereby affecting UV-B-dependent gene expression (Chen et al., 2001), moreover NO was shown to induce *myo*-inositol 1,4,5-trisphosphate synthesis in rat pancreatic cells (Tritsaris et al., 2000; Moustafa et al., 2011). In this work, we investigated the relationships between *myo*-inositol metabolism and NO signaling in the mediation of UV-B induced OS in *Arabidopsis thaliana*, by investigating the response of *atips1* mutants to nitrosative stress and UV-B exposure.

## Materials and Methods

### Plant Material and Treatments

*Arabidopsis thaliana* Columbia 0 wild type (Col-0) and *atips1* mutant (Meng et al., 2009) were transformed with *rogfp2-grx1* (Meyer et al., 2007) by floral-dip as described by Clough and Bent (Clough and Bent, 1998). For OS and plant growth measurements, mutants deficient for *AtERCC1* (At3g05210, UV REPAIR DEFICIENT 7) (Dubest et al., 2004) were used as hypersensitive controls. Sterilized seeds were sown aseptically on half strength MS medium (Duchefa Biochemie, Netherlands) containing 10 g L^-1^ glucose and agar (0,8% w/v). Seedlings were grown in a climate chamber at 24°C under a 16h/8h day/night regime and a light intensity of 3200 lux. 7-day old seedlings were used in all experiments. Plants were treated with 10, 100 μM and 1mM SNP. Incubation and pretreatment with SNP before UV-B irradiation was performed under bright light during 1 h. Plants were irradiated onto solid MS medium by applying 34 and 81 kJ m^-2^ of UV-B as described in (Lytvyn et al., 2010). Impact of SNP and UV-B on seedling growth was measured using the ImageJ software.

### CLSM Measurement of OS

Oxidative stress was measured by Confocal Laser Scanning Microscopy quantification of redox-dependent changes in the fluorescence of roGFP2. Briefly, this approach relies on a modified GFP with cysteine residues that alter its excitation spectrum depending on their oxidation status. Transition of these residues from an oxidized to a reduced state induces a shift in the roGFP2 excitation peak from 405 to 488nm. Mentioned cysteine residues are substrates for GRX and this catalytic reaction mimics the equilibration of cellular glutathione redox buffer [reversible conversion of glutathione from the reduced form (GSH) to the oxidized form (GSSG) Meyer et al., 2007]. Ratiometric analysis of the roGFP2 excitation efficiency at 405 and 488 nm thus reflects the redox status of the cell. Plant **s**amples were examined using a Zeiss LSM 510 META laser scanning confocal microscope equipped with lasers for 405, 488, and 543 nm excitation. When needed, additional staining with 1.5μg ml^-1^ propidium iodide (PI) was performed. Images were collected using a 20x lens (EC Plan-Neofluar 20x/0,5, Zeiss) in multi-track mode with line switching and averaging of two readings. Excitation of reduced roGFP2 and PI was performed in the same track equipped with 488- and 543 nm lasers, respectively. Oxidized roGFP2 was exited at 405 nm in the second track. Fluorescence for both oxidized and reduced roGFP2 was collected with a 505–530 band-pass filter. Power of lasers was adjusted to 20% for excitation of 488 nm and to 40% for 405 nm, respectively. In all experiments calibration procedures with reduction/oxidation of the probe using 10 mM H_2_O_2_ and 10 mM DTT were performed as described in Meyer and Brach (2009). Dynamic measurement of OS development was made under the same conditions with 60 0s intervals in each time series. Estimations of 405/488 nm ratios were performed using Carl Zeiss Laser Scanning Microscope LSM510 Release Version 4.0 SP2. Before calculations of pixel intensity in the area of interest, background level of fluorescence of both 405 and 488 lasers was adjusted to the same basal level. OS development was investigated in five tissues, namely, RT area, EZ, root vessels, and parenchymatous cells of hypocotyls and leaves. In the illustrations excitations of reduced and oxidized roGFP2 are represented with false colors: reduced protein was colored red and oxidized colored blue. All experiments were performed in five and more replications. The results were presented as mean ± SE; statistical analysis was done by Student *t*-test and *P* values less than 0.01 were considered as statistically significant.

### DAB Staining

Staining with 3,3′-diaminobenzidine (DAB) was used as an additional approach to monitor ROS accumulation, notably H_2_O_2_. 0,2% DAB (Sigma, USA) solution filtered with 0,44 μm Millipore membrane was used. Plant samples were incubated for 1h in the staining solution and fixed in 70% ethanol. Samples were then placed in 50% chloral hydrate/glycerol solution (w/v) overnight and mounted for light microscopy.

## Results

### Tolerance of *atips1* Plants to SNP-Induced OS

The main goal of this work was to identify possible cross-talks between *myo*-inositol metabolism and NO signaling. To tackle this issue, we first investigated the sensitivity of Col-0 and *atips1* plants to the NO donor Sodium Nitroprusside (SNP). To this end, we followed the redox status of the roGFP2 sensor (Meyer et al., 2007) in the wild type and mutant backgrounds. roGFP2 was derived from enhanced GFP (EGFP) by introducing two cysteines at positions S147 and Q204, which are located on β-strands and can form a disulfide bond when oxidized. This sensor thus displays redox-dependent changes in its excitation efficiency, the maximal excitation efficiency of the reduced and oxidized forms being 405and 488 nm, respectively (Meyer and Dick, 2010). Thus, the ratio of fluorescence intensity obtained upon illumination at 405 or 488 nm reflects the redox status of the cell cytoplasm, and more specifically of the glutathione pool. Cellular GSH pools are a major non-enzymatic antioxidant system, and relative abundance of the reduced (GSH) and oxidized (GSSG) forms is considered as a reliable indicator of OS level in the cell; in turn, roGFP2 equilibrates with the redox potential of the cellular glutathione buffer via GRX activity (Meyer et al., 2007). In our investigations roGFP2 fused with human Grx1; Grx1-roGFP2 was used to provide higher specificity and sensitivity to the reaction because roGFP reveals slow response to changes in redox potential itself (Gutscher et al., 2008; Meyer and Dick, 2010). In preliminary experiments, we observed that high concentrations of SNP (1 mM and above) induce oxidative/nitrosative stress in *Arabidopsis* (unpublished data), and the same effects were observed in yeasts and insect cells (Lushchak and Lushchak, 2008; Lozinsky et al., 2012). We thus chose to apply 1 mM SNP treatment to follow nitrosative/OS development in our lines. We followed changes in the redox status over time in above-ground and below-ground tissues and in various cell types.

In the wild-type, we observed differences in the basal cellular redox status depending on the tissues: cells of the RT, vessels, and aerial tissues being more oxidized than cells of the elongation zone (EZ). The basal redox status was similar in wild-type and *atips1* mutant plants in most cell types except in the vasculature and RT in which basal oxidation levels were slightly higher in the mutant (**Figures 1.1–1.4**). Upon SNP treatment, we did not observe any OS development in leaves or hypocotyls (**Figure 1.4**). By contrast, root cells of the RT and EZ showed clear OS development in the wild-type, but not in the mutant where the redox status remained stable (**Figures 1.1–1.3**). In vascular tissues, OS levels remained unchanged in *atips1* but increased in the wild-type until they reached higher levels than in the mutant (**Figure 1.3**). Overall, the *atips1* was thus found to be tolerant to the nitrosative effects of SNP in all root cell types.

**FIGURE 1:**
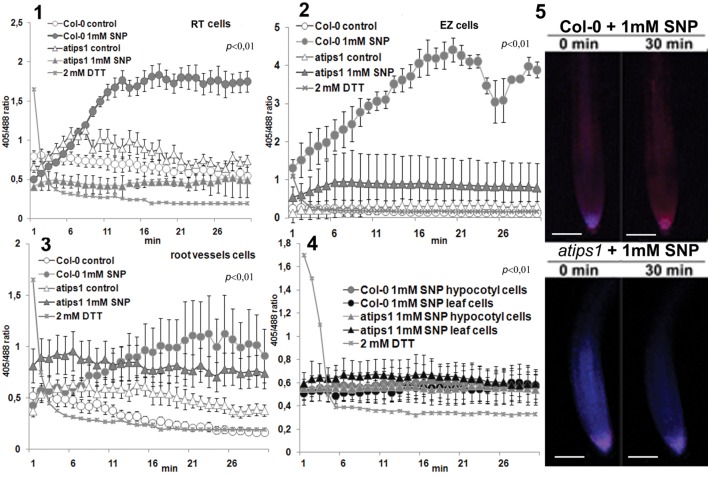
**Dynamics of roGFP2 oxidation in RT cells **(1)**, root EZ cells **(2)**, root vessels **(3)**, hypocotyl/leaf cells of Col-0 (4) and *atips1* under 30 min treatment with 1 mM SNP **(5)**.** **(5)** Confocal overlay of the roGFP2 emission under 1 mM SNP treatment in the first and last time points. Notice almost intact redox status in the mutant, swift OS development in wild type since the initial treatment point and high initial oxidation level in RT cells. “2 mM DTT” in the legend displays calibration control of the method: sample oxidized with 10 mM H_2_O_2_ and subjected to 2 mM DTT treatment just before mesurment. Bar – 100 μM.

### Redox Effects of SNP are Highly Tissue Specific and are Altered in the *atips1* Mutant

**FIGURE 2:**
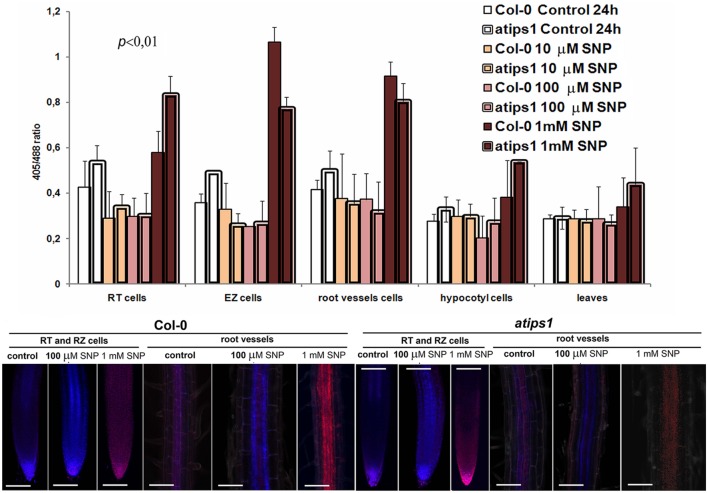
**Oxidizing and reducing tissue-specific effects of 24 h treatment of Col-0 and *atips1* seedlings with different SNP concentrations.** Lower panel displays an example of confocal overlay of roGFP2 oxidation/reduction in root tissues. Bar – 100 μM.

To determine the sensitivity of various cell types to different amounts of NO-donor, an investigation of OS level was performed after 24 h of SNP treatment. Analysis was performed at late time points because the cellular redox status at the earlier time points was highly heterogeneous (not shown). This observation can be explained by the variability of the initial starting oxidation level in different plants that was leveled within 24 h treatment. SNP had either oxidizing or reducing action depending on the applied concentration (**Figure 2**). Ten and 100 μM SNP did not significantly modify the redox status of roGFP in the wild type, but induced significant reduction in root cells of the *atips1* mutants both in the RT and in the EZ cells. Other tissues were characterized by weak differences with a tendency to reduction in treated cells. After treatment with 1 mM SNP, both Col-0 and *atips1* plants exhibited near similar OS development in roots, but statistically significant OS development was also detected in mutant plants under the same conditions in hypocotyls and leaves.

Together, obtained data show that SNP has highly dose-dependent effects, low doses leading to a reduction of the cytoplasm whereas high doses induce OS. Furthermore, our results suggest that a signaling and/or metabolic link between *myo*-inositols and NO-induced OS exists in plant cells, and that this interrelation is strongly tissue-specific.

### Prevention of UV-B Induced Oxidation by Pretreatment with SNP is More Effective in *atips1*

**FIGURE 3:**
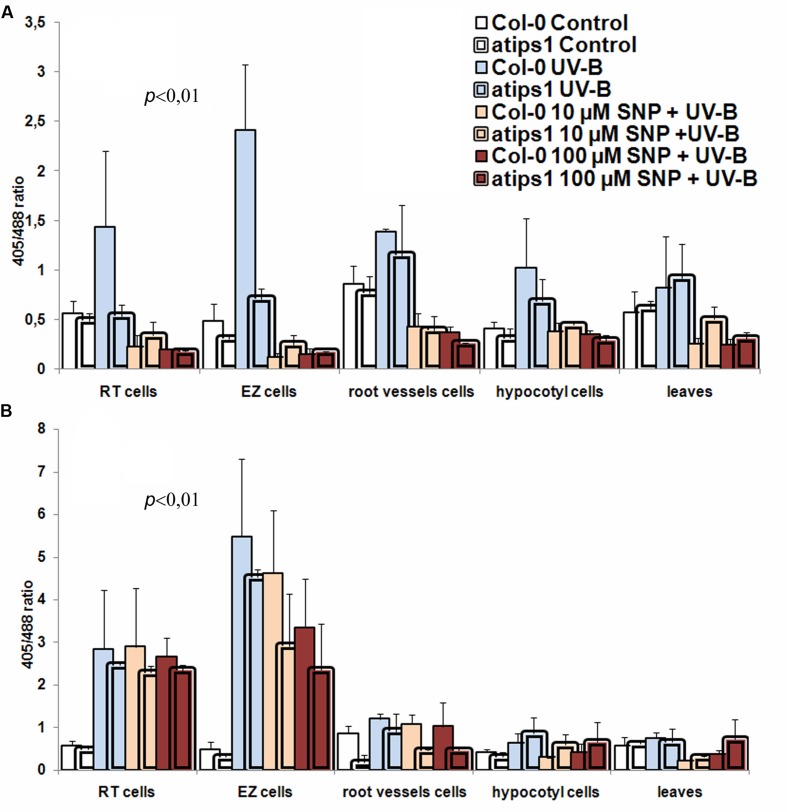
**Oxidative stress development and protective effects of SNP in plants irradiated with 34 kJ m^-2^**(A)** and 81 kJ m^-2^**(B)** of UV-B**.

Because NO signaling has been involved in the cellular response to UV-B, we next investigated the interplay between *myo*-inositol accumulation and NO in this process. Determination of OS levels immediately after plants irradiation with 34 and 81 kJ m^-2^ of UV-B did not reveal statistically relevant changes compared to untreated plants. Therefore, UV-B oxidation effects were probed after 24h of treatment. Obtained results are summarized on **Figure 3**.

In the wild-type, UV-B irradiation induced OS in all analyzed tissues, root tissues, and particularly the RT and EZ being the most sensitive, and vascular cells the most tolerant. Hypocotyl cells showed some UV-B dose-dependent changes of oxidation levels that were more pronounced in the wild type, whereas in mesophyll cells, UV-B irradiation did not cause statistically significant OS. At 34 kJ.m^-2^, *atips1* mutants displayed greater resistance to the harmful action of UV-B. Indeed in the wild type, UV-B irradiation caused a 2,5 fold and 4,9 fold increase of OS level in RT cells and cells of the EZ, respectively, whereas this increase was absent in the RT and only about twofold in the EZ in the *atips1* mutant. These results suggest that *atips1* is tolerant to the oxidative effect of UV-B.

Pretreatment of wild-type plants with SNP revealed clear anti-oxidizing effects under UV-B irradiation (**Figure 3A**). Indeed, incubation of Col-0 plants with both 10 and 100 μM SNP before irradiation at 34 kJ m^-2^ resulted in a sharp reduction of OS development: OS could no longer be observed in any of the tested cell types. The reducing action of SNP was so effective that oxidation levels in pretreated irradiated samples were even lower than in unirradiated plants. A similar response was observed in *atips1* plants (**Figure 3B**), but the anti-oxidant effect of SNP treatment was less pronounced in *atips1* due to the higher basal tolerance of the mutant to UV-B. Pretreatment of above-ground tissues led to similar reducing patterns, but had low intensity (**Figure 3A**).

Generally, pretreatment with SNP had no reducing effects in most tissues of wild type plants irradiated at 81 kJ.m^-2^. By contrast, a statistically significant protective impact of NO was observed in the EZ and root vessels cells of *atips1* pretreated with 100 μM SNP. Indeed, roGFP2 in the cells of EZ was in 1,9 times more reduced after irradiation than in untreated plants. Cells of *atips1* vessels showed increased OS in response to this dose of UV-B compared to the wild type, due to the lower basal reduction of the reporter. In this cell type, pre-treatment with SNP again resulted in a protective effect against the oxidative effect of NO (**Figure 3B**). Together, results obtained after irradiation with higher UV-B dose support the notion that *myo*-inositol metabolism affects UV-B-induced OS.

**FIGURE 4:**
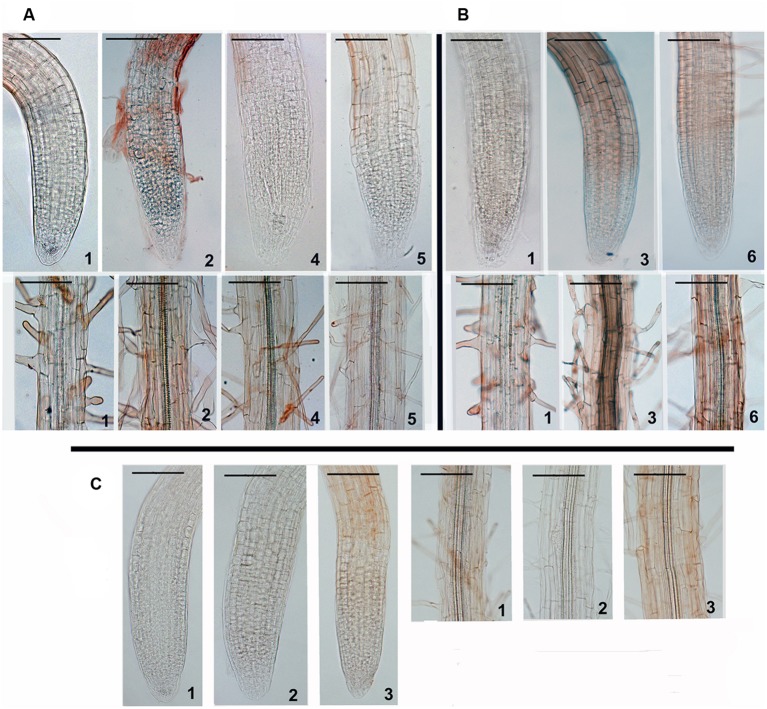
**Synergistic impact of SNP and UV-B on the intracellular level of hydrogen peroxide in Col-0 **(A)**, *atips1***(B)**, and *atercc1***(C)** plants (DAB staining): 1-control; 2- irradiation with 34 kJ m^-2^ UV-B; 3- irradiation with 81 kJ m^-2^ UV-B; 4- pretreatment with 10 μM SNP before irradiation with 34 kJ m^-2^ UV-B; 5- pretreatment with 100 μM SNP before irradiation with 34 kJ m^-2^ UV-B; 6- pretreatment with 100 μM SNP before irradiation with 81 kJ m^-2^ UV-B.** Brown color intensity reflects H_2_O_2_ level in the cell.

Quantitative features of oxidative UV-B impact and protective effects of SNP were confirmed by qualitative staining of hydrogen peroxide as one of the main messenger ROS molecules (Van Breusegem et al., 2008). In these experiments, the ultraviolet sensitive *AtERCC1* mutant was used as an additional control. In this mutant, hydrogen peroxide accumulation was very low and showed little variation under all combinations of treatments (**Figure 4C**). Pretreatment of both Col-0 and *atips1* seedlings with 100 μM SNP lead to a decrease in H_2_O_2_ accumulation induced by 34 kJ m^-2^ of UV-B in root tissues, but only in mutant plants was this effect still observed after irradiation with 81 kJ m^-2^.

To investigate the physiological relevance of the above-described observations, we monitored the effect of combined UV-B and SNP treatment on organ growth. Three-day-old Col-0, *atips1*, and *atercc1* seedlings subjected to treatment with different concentration of SNP, UV-B irradiation and combined SNP pretreatment and UV-B were grown for four days for time-course analysis of roots and hypocotyls growth. Control *atercc1* plants demonstrated the highest sensitivity to SNP: all inhibited both root and hypocotyls growth (**Figure 5.3**). In addition Col-0 and *atips1* seedlings treatment with 100 μM SNP also led to a small but statistically significant suppression of root growth (**Figures 5.1,2**). We therefore used 10 μM SNP as working concentration for combination with UV-B irradiation in growth experiments. Inhibitory impact of irradiation and protective effect of SNP were observed upon plant treatment with 34 kJ m^-2^ of UV-B, but it was not pronounced or statistically valid, probably because of the low cytotoxity of this UV-B dose. 81 kJ m^-2^ of UV-B led to an almost complete arrest of seedlings growth in all lines. However, pretreatment with 10 μM SNP resulted in partial recovery of seedlings growth (roots of Col-0, and roots and hypocotyls of *atips1*) and this effect was also more pronounced in *atips1* plants (**Figure 5.4**).

**FIGURE 5:**
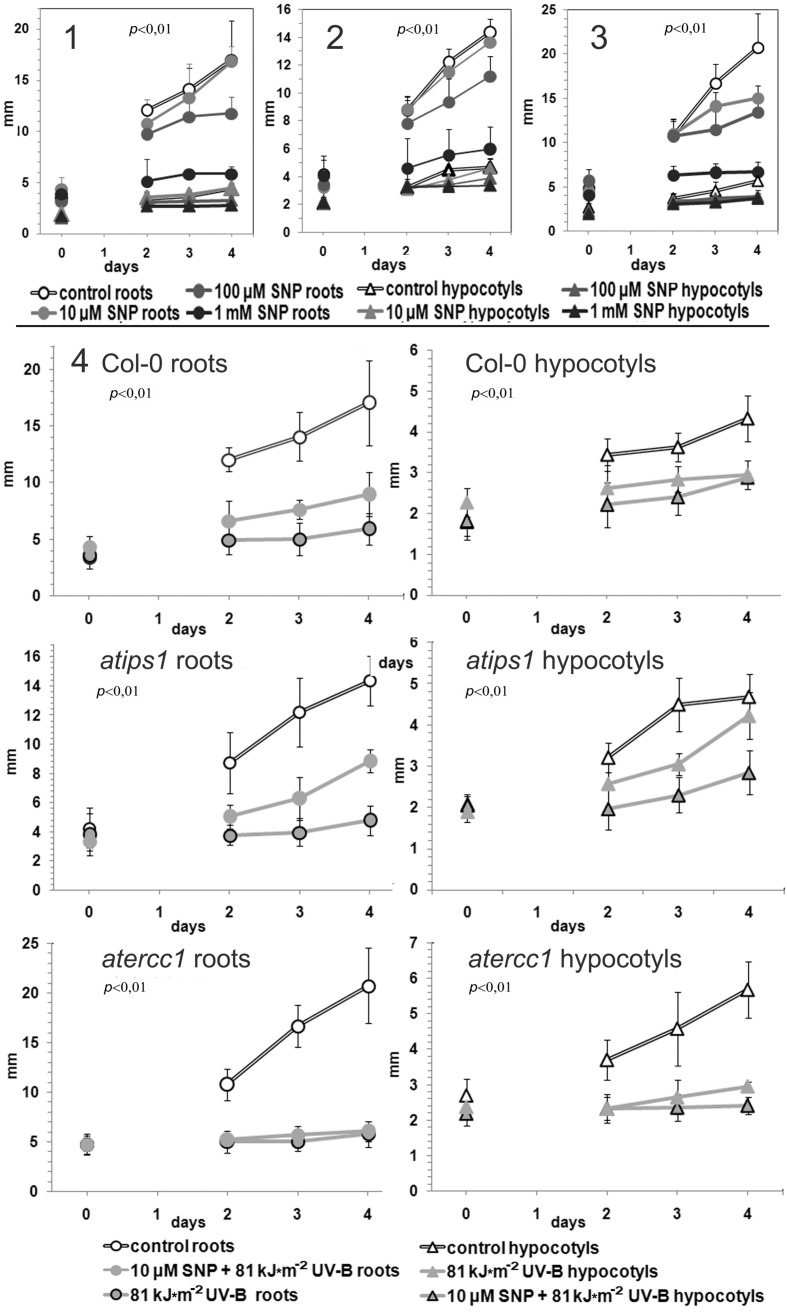
**Growth rates of wild type plants **(1)**, and *atips1***(2)**, *atercc1***(3)** mutants treated with different concentrations of SNP; and all mentioned lines that were irradiated with 81 kJ m^-2^ UV-B alone and with 10 μM SNP pretreatment **(4)****.

## Discussion

To summarize, our results allowed us to define several aspects of plant response to SNP and UV exposure. We demonstrate that NO has highly tissue-specific effects, and that its cellular impact is modified according to *myo*-inositol accumulation. In the wild-type, RT and EZ cells were extremely sensitive to oxidation caused by high SNP concentrations, and this effect was completely abolished by *myo*-inositol deficiency; vascular cells were less sensitive to SNP but *atips1* mutants were also more tolerant than the wild-type; after 24 h of treatment similar oxidation patterns were observed in root tissues of both wild type and mutant plants, but mutants also showed OS development in underground tissues (**Figures 1** and **3**); SNP in low (10 and 100 μM) concentrations had a protective effect toward UV-B (**Figures 3** and **4**); *atips1* mutant was both more tolerant to the oxidative impact of UV-B and more perceptive to the protective action of NO under UV-B irradiation (**Figures 3–5**). Altogether these findings strongly suggest direct influence of *myo*-inositol metabolism on the NO-mediated stress signaling.

The validity of these results is supported by the use of a redox-sensitive GFP probe that is an extremely sensitive tool allowing accurate *in vivo* analysis of redox fluctuations over time. Because the roGFP2 sensor used in this study was fused to Grx1, its redox status reflects the redox status of the cellular glutathione pool (Gutscher et al., 2008; Meyer and Dick, 2010). Using the roGFP2 probe is appropriate in the investigations of NO impact on the cellular redox status because of the tight interrelation between GSH homeostasis and NO levels. Indeed, in neuronal cells, GSH decrease was shown to cause protein nitration, *S*-nitrosylation, DNA breaks (Aquilano et al., 2011a) and to induce cell death in response to neurotrophic doses of NO (Canals et al., 2001, 2003). These data support the idea that GSH is the most important buffer of NO toxicity in neuronal cells, and cellular redox buffering controlled by GSH makes neuronal cells susceptible to endogenous physiological flux of NO (Aquilano et al., 2011a,b). In addition, close crosstalk between stressful oxidative and nitrosative signaling is generally accepted. Multiple lines of evidence confirm that ROS and RNS, namely, eroxyinitrite (ONOO^-^), nitrogen dioxide (.NO_2_), dinitrogen trioxide (N_2_O_3_) and *S*-nitrosoglutathione) in plants act together to modulate cellular responses to environmental stimuli. First RNS and ROS pools are linked both by direct chemical interaction between ROS and RNS and by interlinking molecules such as polyamines. Notably, GSNO is recognized as a key element in the interplay between the ROS and RNS metabolisms: GSNO is considered as an intracellular NO depot which is formed by NO’s *S*-nitrosylation reaction with reduced glutathione (GSH; Corpas and Barroso, 2013). Second, there is significant overlap between the ROS/RNS-responsive gene networks and ROS/RNS-responsive proteins. Finally, ROS- and RNS-based stressful post-translational modifications of the proteins also have similarities (Molassiotis and Fotopoulos, 2011; Corpas and Barroso, 2013).

Our data highlighting oxidative impact of NO excess is consistent with the previously reported harmful effects of high NO levels in plant cells. NO has been shown to induce membrane damaging, DNA fragmentation, and reduction of photosynthesis and respiration; moreover, NO concentrations higher than 10 μM were shown to impair leaf expansion as well as shoot and root growth, and to induce changes in thylakoid viscosity, to impair photosynthetic electron transport, and to cause DNA damage and cell death (summarized in (Siddiqui et al., 2011)). Indeed, 1 mM SNP was an extremely cytotoxic amount and this treatment led to both clear OS development and total inhibition of root and hypocotyls growth in all examined *Arabidopsis* lines. However, under lower concentration (10 and 100 μM SNP) treatments two distinct (sort- and long-term) plant responses were observed. Short term (30 min) incubation with mentioned concentrations had no reliable effects on redox levels (not shown), and even induced mild reduction in root tissues (especially in *atips1*) within 24 h of treatment (**Figure 2**); but after four days following treatment statistically valid inhibition of root growth was observed in all lines (**Figures 5.1–5.3**). Notably, in *atercc1* the same effect was observed even after treatment with 10 μM SNP (**Figure 5.3**). ERCC1, is involved in the removal of non-homologous tails in homologous recombination (Dubest et al., 2002, 2004) and recombination-related DNA repair (Hefner et al., 2003), it is thus logical to suggest mentioned processes as one of the downstream targets of NO signaling and/or RNS impact.

One of the main findings discovered in this work is that the protective NO action against UV-B irradiation varies according to cell type. Indeed in our experiments, UV-B overexposure led to dose-dependent OS development that showed clear tissue specificity: RT cells were more sensitive to UV-B treatment than EZ cells or leaf parenchyma for example, consistent with the activation of anti-oxidant system in photosynthetic tissues. Interestingly, *atips1* was more resistant to NO induced OS than the wildtype, in line with the previous observation that this mutant is tolerant to OS induced by paraquat (Meng et al., 2009). OS generated by UV-B exposure also caused increased H_2_O_2_ production (**Figures 4A2, B3**), and plant growth inhibition. However, plant pretreatment with 100 μM SNP led to leveling and even decreasing to lower then initial redox state in all examined tissues of Col-0 and *atips1* under 34 kJ m^-2^ irradiation (**Figure 3A**). NO pre-treatment could not prevent UV-B induced OS in wild-type plant, but was able to reduce roGFP2 oxidation in the *atips1* background exposed to extreme (81 kJ m^-2^) doses of UV-B (**Figure 3B**). Again, this finding provides evidence for the greater tolerance of the *atips1* mutant to OS: in this background, NO pre-treatment likely activated pathways involved in the protection against OS, thereby resulting in lower OS after UV-B treatment. Moreover, analysis of plant growth after UV-B exposure confirmed these hypotheses: the protective effect of NO pretreatment was more pronounced in *atips1* where in contrast to Col-0 it was observed both in roots in hypocotyls (**Figure 5.4**). Whether contrasting *myo*-inositol levels can account for the tissue specificity of NO response remains an open question. Indeed, *myo*-inositol quantification has been performed in leaves of the *atips1* mutant and has revealed a drastic reduction of the *myo*-inositol content. *Myo*-inositol accumulation is likely to be also severely affected in roots since the atips1 mutant has a root growth phenotype that can be rescued by addition of *myo*-inositol to the growth medium (Meng et al., 2009). These findings support the view that the higher tolerance of *atips1* to NO is indeed attributable to the reduction *myo*-inositol accumulation, but available methods for *myo*-inositol quantification do not allow to correlate the response of individual cell types to their *myo*-inositol levels.

A number of reports shed light on the underlying mechanisms that may account for the protective role of NO toward UV-B cytotoxicity. In plants, NO is involved in the tolerance to salinity, drought, ultraviolet, temperature, and heavy metals stresses (Siddiqui et al., 2011). Abiotic stresses provoke changes in NO levels directly or via ROS and hormonal mediation that, in turn, leads to activation of antioxidant enzymes expression and elimination of superoxide anions and lipid radicals (Siddiqui et al., 2011). Nitric oxide synthase (NOS) activity was shown to be significantly increased by UV-B irradiation, suggesting that NO may act as a secondary messenger under UV-B irradiation (Yemets et al., 2015). In plants, action of exogenous NO (namely, using SNP as a donor) under UV-B irradiation was shown to allow photosystem II protection and ROS scavenging (Shi et al., 2005; Wang et al., 2008). Described scavenging mechanisms include regulation of GSH accumulation, elimination of superoxide anions, increase of superoxide dismutase, catalase and peroxidase (Xue et al., 2007) as well ascorbate peroxidase (Santa-Cruz et al., 2014) activities; up-regulation of antioxidant heme oxygenase (Santa-Cruz et al., 2010) and decrease of nitrogenase activity (Xue et al., 2011). Recently, complex analysis of synergistic impact of SNP and UV-B in lettuce seedlings revealed that the above-mentioned pretreatment resulted in enhanced antioxidant enzyme activities, total phenolic concentrations, antioxidant capacity and expression of phenylalanine ammonia lyase; alleviation of chlorophylls, carotenoid, gibberellic and indole-3-acetic acid content inhibition, as well as in decrease of abscisic and salicylic acids (SA), malondialdehyde, hydrogen peroxide and superoxide anion (Esringu et al., 2015). In addition, earlier we have shown that exogenous NO protects organization of plant microtubules against disrupting effects of UV-B (Krasylenko et al., 2012).

NO-dependent changes in H_2_O_2_ accumulation were another notable finding of this work. As a rule, root tissues of intact plants were characterized by near undetectable H_2_O_2_ which was dramatically increased following irradiation with both UV-B doses (aboveground tissues were not investigated because of high basal levels of H_2_O_2_) (**Figures 4A1,B1**). SNP pretreatment of wild type and mutant plants before exposure to 34 kJ m^-2^ of UV-B completely abolished H_2_O_2_ production (**Figures 4A4,A5**). But only pretreated mutant plants demonstrated a clear decrease of H_2_O_2_ production, when irradiated with a higher UV-B dose (**Figures 4B3,B6**). This result is consistent with the above-mentioned role of exogenous NO in the regulation of catalase and peroxidase activity, and the finding that inhibition of NOS activity leads to similar consequences under UV-B irradiation (Kim et al., 2010). It is worth noting that in *atercc1* plants the effects of UV-B irradiation were totally different from Col-0 and *atips1*. Indeed, UV-B impact had no effects on H_2_O_2_ accumulation *in atercc1* compared to control (**Figures 4C1–4C3**). Since this line is hypersensitive to UV-B (**Figure 5.4**), we can assume that H_2_O_2_ may also play a signaling role in the UV-B response. Indeed, NO and H_2_O_2_ were identified as important early upstream signaling components which regulate expression of different sets of genes involved in defense and tolerance to UV-B radiation (Mackerness et al., 2001). The production of both molecule is known to be activated by various stresses and their synergistic signaling activity is cross-regulated with abscisic, jasmonic, salicylic acid and ethylene (Neill et al., 2002). H_2_O_2_ was proposed as a signal molecule in the mediation of excess excitation energy stress in *Arabidopsis* and this mediation is linked with changes in the redox state of the cellular glutathione pool (Mullineaux et al., 2000). However, the above-described phenomenon of more pronounced NO influence effects on H_2_O_2_ homeostasis in *atips1* denotes the unknown interplay between *myo*-inositol and NO signaling pathways in stress-related regulation of H_2_O_2_ level.

The most debatable finding of this work is the unexpected resistance of *atips1* plants to NO and UV-B induced OS as well as its enhanced receptivity to protective effects of exogenous NO. Interestingly, resistance of the mutant is stimuli-specific: *atips1* mutants are tolerant to paraquat (Meng et al., 2009), but previously, using the same roGFP2-GRX expressing lines, we observed that *atips1* plants have considerably higher sensitivity to OS induced by SA (Lytvyn et al., 2011). These contrasting behaviors probably relate to the conditional cell death phenotype observed in *atips1*: exposure to long days or high light intensity induces a peak in SA production, leading to lesion formation in the mutant (Meng et al., 2009; Donahue et al., 2010). Thus this mutant shows enhanced basal tolerance to OS, but SA accumulation elevates OS above a threshold leading to cell death.

In animal cells, the protective role of *myo*-inositols toward exposure to UV-B is well documented. Indeed, inositol hexaphosphate (InsP6) prevents activation of activator protein-1 (AP-1) and NF-kappaB as well as phosphorylation of extracellular signal-regulated protein kinases (Erks) and c-Jun NH2-terminal kinases (JNKs) in response to UV-B, thereby avoiding UV-B-induced carcinogenesis (Chen et al., 2001). Additionally, inositol was shown to be involved in the maintenance of cell volume homeostasis in human keratinocytes under UV-B irradiation (Warskulat et al., 2004). Finally, UV-B irradiation may also result in *myo*-inositol decrease in rabbit cornea and lens cells, pointing to the interrelation between UV-B response, *myo*-inositol and osmotic regulation (Risa et al., 2005; Tessem et al., 2006). Whether similar mechanisms exist in plants remains unknown. However, IPS1 was shown to be down-regulated under UV-B irradiation in maize (Chen et al., 2001; Content et al., 2003) and *myo*-inositol accumulation was shown to vary upon UV-B exposure (Casati et al., 2011). We suggest that *myo*-inositol deficiency may activate other protective mechanisms against UV. Indeed, we have shown previously in (Meng et al., 2009) that *atips1* mutants grown under short day cultivation (so without cell death) are tolerant to OS. Our hypothesis is that *myo*-inositol deficiency causes a basal stress level that activates protective mechanisms. This priming of plant defense may enhances their tolerance to stress up to a certain threshold, and, beyond this threshold, cell death is induced.

Finally, the increased resistance of *atips1* to OS may be connected not only with the catalytic function of AtIPS1, but also with the involvement of this enzyme in the regulation of gene expression. Indeed the AtIPS1 protein was identified as a interacting partner of the histone methyltransferase *Arabidopsis* Trithorax-Related Protein 5 (ATXR5) (Meng et al., 2009), and can inhibit its activity to control its own expression (Latrasse et al., 2013). This regulatory function may also occur at other loci, possibly including genes involved in OS response. Transcriptomic analysis of gene expression profiles of *atips1* plants grown under long day conditions revealed upregulation of 52 genes potentially involved in the processes associated with OS mediation (Meng et al., 2009), whether this effect is due to direct regulation of their expression by AtIPS1 or to indirect effects caused by modified *myo*-inositol accumulation remains to be established. Enhanced protective effect of NO in plants deficient for AtIPS1 sheds light on the relationship between this enzyme functions and regulation of expression and/or activity of antioxidant systems upon exposure to UV-B in plant cell.

**FIGURE 6:**
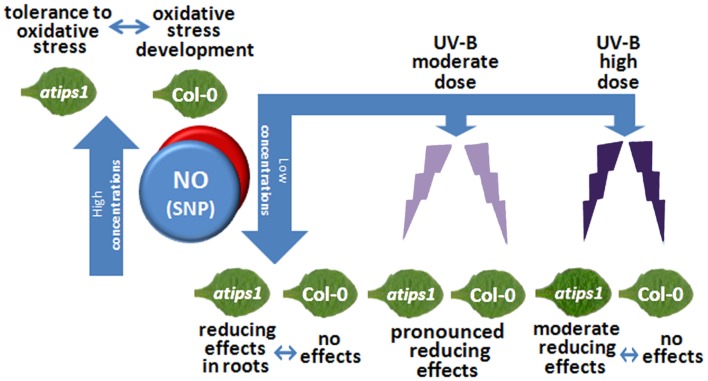
**Summarize dose-dependent redox behavior of Col-0 and *atips1* plants under SNP treatment and UV-B irradiation.** IPS1-difficient plants demonstrate both tissue-specific tolerance to NO excess and higher receptivity to protective NO effect under ultraviolet irradiation.

Presented results are a prerequisite for further study of the role of the 1-L-*myo*-inositol-1-phosphate 1 in the mediation of external abiotic impacts, including UV-B. On this step the interrelations between *myo*-inositol metabolism, NO signaling, and OS mediation became evident under both harmful influence of NO excess (insensitivity of underground tissues of the mutant to nitrosative impact comparing to wild type) and variability of tissues-specific reduction/oxidation answers of the *atips1* and Col-0 plants to different concentrations of NO donor. Higher tolerance to UV-B action and increased receptivity to protective NO effects of *atips1* are the arguments of mentioned interrelation too (summarized in **Figure 6**). Unraveling the underlying molecular mechanisms will be a challenging goal for future investigation.

## Author Contributions

DL, formulation of the work conception and experimental design. Performing of the experiments, data analysis, and manuscript preparing. CR, obtaining of transgenic plants, data analysis, and interpretation, manuscript preparing, and revising it critically for important intellectual content. AY, experimental design, data analysis, and interpretation, manuscript preparing and final approval of the version to be published. CB, formulation of the work conception, obtaining of transgenic plants, data analysis, and interpretation. YB, contributions to the work conception, data analysis, interpretation of results, and final approval of the version to be published.

## Conflict of Interest Statement

The authors declare that the research was conducted in the absence of any commercial or financial relationships that could be construed as a potential conflict of interest.

## Abbreviations

EZ: root elongation zone
GRX1: glutaredoxin
GSNO: *S*-nitrosoglutahtione
IPS1: inositol-3-phosphate synthase 1
NO: nitric oxide
OS: oxidative stress
roGFP2: reduction–oxidation-sensitive green fluorescent protein 2
RNS: reactive nitrogen species
ROS: reactive oxygen species
RT: root tip area
SNP: sodium nitropusside
